# Hints Worth Remembering

**Published:** 1892-11

**Authors:** George A. Maxfield


### HINTS WORTH REMEMBERING.

BY GEO. A. MAXFIELD, D.D.S.

To remove the stains of tincture of iodine, from either the hands or napkins, apply strong ammonia. The spots will immediately come out clear.

The stains of nitrate of silver, from either the hands or napkins, can be easily removed. First cover the spots with tincture of iodine, wait a few moments, then apply strong ammonia, and rub well.

In using argenti nitras in treating children's teeth one may accidentally get a few grains of the powder on his hands and not discover it till the hands are washed, when the black stains will be well set. By proceeding as above, they will disappear at once.

